# The ENIGMA‐Epilepsy working group: Mapping disease from large data sets

**DOI:** 10.1002/hbm.25037

**Published:** 2020-05-29

**Authors:** Sanjay M. Sisodiya, Christopher D. Whelan, Sean N. Hatton, Khoa Huynh, Andre Altmann, Mina Ryten, Annamaria Vezzani, Maria Eugenia Caligiuri, Angelo Labate, Antonio Gambardella, Victoria Ives‐Deliperi, Stefano Meletti, Brent C. Munsell, Leonardo Bonilha, Manuela Tondelli, Michael Rebsamen, Christian Rummel, Anna Elisabetta Vaudano, Roland Wiest, Akshara R. Balachandra, Núria Bargalló, Emanuele Bartolini, Andrea Bernasconi, Neda Bernasconi, Boris Bernhardt, Benoit Caldairou, Sarah J.A. Carr, Gianpiero L. Cavalleri, Fernando Cendes, Luis Concha, Patricia M. Desmond, Martin Domin, John S. Duncan, Niels K. Focke, Renzo Guerrini, Khalid Hamandi, Graeme D. Jackson, Neda Jahanshad, Reetta Kälviäinen, Simon S. Keller, Peter Kochunov, Magdalena A. Kowalczyk, Barbara A.K. Kreilkamp, Patrick Kwan, Sara Lariviere, Matteo Lenge, Seymour M. Lopez, Pascal Martin, Mario Mascalchi, José C.V. Moreira, Marcia E. Morita‐Sherman, Heath R. Pardoe, Jose C. Pariente, Kotikalapudi Raviteja, Cristiane S. Rocha, Raúl Rodríguez‐Cruces, Margitta Seeck, Mira K.H.G. Semmelroch, Benjamin Sinclair, Hamid Soltanian‐Zadeh, Dan J. Stein, Pasquale Striano, Peter N. Taylor, Rhys H. Thomas, Sophia I. Thomopoulos, Dennis Velakoulis, Lucy Vivash, Bernd Weber, Clarissa Lin Yasuda, Junsong Zhang, Paul M. Thompson, Carrie R. McDonald, ENIGMA Consortium Epilepsy Working Group, Eugenio Abela, Julie Absil, Sophia Adams, Saud Alhusaini, Marina Alvim, Simona Balestrini, Benjamin Bender, Felipe Bergo, Tauana Bernardes, Anna Calvo, Mar Carreno, Andrea Cherubini, Philippe David, Esmaeil Davoodi‐Bojd, Norman Delanty, Chantal Depondt, Orrin Devinsky, Colin Doherty, Wendy Caroline França, Leticia Franceschet, Derrek P. Hibar, Akari Ishikawa, Erik Kaestner, Soenke Langner, Min Liu, Laura Mirandola, Jillian Naylor, Mohammad‐reza Nazem‐Zadeh, Terence J. O'Brien, Letícia F. Ribeiro, Mark Richardson, Felix Rosenow, Mariasavina Severino, Chen Shuai, Domenico Tortora, Felix von Podewils, Sjoerd B. Vos, Jan Wagner, Guohao Zhang

**Affiliations:** ^1^ Department of Clinical and Experimental Epilepsy UCL Queen Square Institute of Neurology London UK; ^2^ Chalfont Centre for Epilepsy Bucks UK; ^3^ Department of Molecular and Cellular Therapeutics The Royal College of Surgeons in Ireland Dublin Ireland; ^4^ Center for Multimodal Imaging and Genetics University of California San Diego La Jolla California USA; ^5^ Centre for Medical Image Computing, Department of Medical Physics and Biomedical Engineering University College London London UK; ^6^ UCL Queen Square Institute of Neurology London UK; ^7^ Department of Neuroscience Istituto di Ricerche Farmacologiche Mario Negri IRCCS Milan Italy; ^8^ Neuroscience Research Center, Department of Medical and Surgical Sciences University “Magna Græcia" of Catanzaro Catanzaro Italy; ^9^ Institute of Neurology University “Magna Græcia" of Catanzaro Catanzaro Italy; ^10^ Neuroscience Institute University of Cape Town Cape Town South Africa; ^11^ Department of Biomedical, Metabolic, and Neural Sciences University of Modena and Reggio Emilia Modena Italy; ^12^ Neurology Unit OCB Hospital, AOU Modena Modena Italy; ^13^ Department of Psychiatry University of North Carolina Chapel Hill North Carolina USA; ^14^ Department of Computer Science University of North Carolina Chapel Hill North Carolina USA; ^15^ Department of Neurology Medical University of South Carolina Charleston South Carolina USA; ^16^ Support Center for Advanced Neuroimaging University Institute of Diagnostic and Interventional Neuroradiology, Inselspital, Bern University Hospital, University of Bern Bern Switzerland; ^17^ Boston University School of Medicine Boston Massachusetts USA; ^18^ Magnetic Resonance Image Core Facility Institut d'Investigacions Biomèdiques August Pi i Sunyer (IDIBAPS), Universitat de Barcelona Barcelona Spain; ^19^ Radiology Department of Center of Image Diagnosis Hospital Clinic de Barcelona Barcelona Spain; ^20^ Neurology Unit USL Centro Toscana, Nuovo Ospedale Santo Stefano Prato Italy; ^21^ Neuroimaging of Epilepsy Laboratory Montreal Neurological Institute, McGill University Montreal Québec Canada; ^22^ McConnell Brain Imaging Center Montreal Neurological Institute, McGill University Montreal Québec Canada; ^23^ Neuroscience Institute of Psychiatry, Psychology and Neuroscience London UK; ^24^ School of Pharmacy and Biomolecular Sciences The Royal College of Surgeons in Ireland Dublin Ireland; ^25^ FutureNeuro SFI Research Centre Dublin Ireland; ^26^ Department of Neurology and Neuroimaging Laboratory University of Campinas – UNICAMP Campinas São Paulo Brazil; ^27^ Instituto de Neurobiología Universidad Nacional Autónoma de México Querétaro Mexico; ^28^ Department of Radiology Royal Melbourne Hospital, University of Melbourne Melbourne Victoria Australia; ^29^ Functional Imaging Unit, Department of Diagnostic Radiology and Neuroradiology University Medicine Greifswald Greifswald Germany; ^30^ University Medicine Göttingen Clinical Neurophysiology Göttingen Germany; ^31^ Pediatric Neurology, Neurogenetics and Neurobiology Unit and Laboratories Children's Hospital A. Meyer‐University of Florence Florence Italy; ^32^ The Wales Epilepsy Unit, Department of Neurology University Hospital of Wales Cardiff UK; ^33^ Cardiff University Brain Research Imaging Centre, School of Psychology Cardiff University Cardiff UK; ^34^ Department of Neurology Austin Health Heidelberg Victoria Australia; ^35^ Florey Department of Neuroscience and Mental Health University of Melbourne Victoria Australia; ^36^ Imaging Genetics Center Mark and Mary Stevens Institute for Neuroimaging and Informatics, Keck School of Medicine, University of Southern California Marina del Rey California USA; ^37^ Kuopio University Hospital Member of EpiCARE ERN Kuopio Finland; ^38^ Institute of Clinical Medicine Neurology, University of Eastern Finland Kuopio Finland; ^39^ Institute of Systems, Molecular and Integrative Biology University of Liverpool Liverpool UK; ^40^ The Walton Centre NHS Foundation Trust Liverpool UK; ^41^ Department of Psychiatry University of Maryland School of Medicine Baltimore Maryland USA; ^42^ Department of Neuroscience, Central Clinical School Monash University Melbourne Victoria Australia; ^43^ Functional and Epilepsy Neurosurgery Unit, Neurosurgery Department Children's Hospital A. Meyer‐University of Florence Florence Italy; ^44^ Department of Neurology and Epileptology Hertie Institute for Clinical Brain Research, University Hospital Tübingen Tübingen Germany; ^45^ 'Mario Serio' Department of Clinical and Experimental Medical Sciences University of Florence Florence Italy; ^46^ Cleveland Clinic Neurological Institute Cleveland Ohio USA; ^47^ Department of Neurology New York University School of Medicine New York New York USA; ^48^ Department of Diagnostic and Interventional Neuroradiology University Hospitals Tübingen Tübingen Germany; ^49^ Department of Clinical Neurophysiology University Hospital Göttingen Goettingen Germany; ^50^ Montreal Neurological Institute and Hospital McGill University Montreal Québec Canada; ^51^ EEG and Epilepsy Unit Geneva Switzerland; ^52^ The Florey Institute of Neuroscience and Mental Health Austin Campus Heidelberg Victoria Australia; ^53^ Department of Neuroscience Monash University Melbourne Victoria Australia; ^54^ Alfred Health Melbourne Victoria Australia; ^55^ Radiology and Research Administration Henry Ford Health System Detroit Michigan USA; ^56^ School of Electrical and Computer Engineering College of Engineering, University of Tehran Tehran Iran; ^57^ South African Medical Research Council Unit on Risk & Resilience in Mental Disorders, Dept of Psychiatry & Neuroscience Institute University of Cape Townon Risk & Resilience in Mental Disorders Cape Town South Africa; ^58^ Pediatric Neurology and Muscular Diseases Unit IRCCS Istituto 'G. Gaslini' Genova Italy; ^59^ Department of Neurosciences, Rehabilitation, Ophthalmology, Genetics, Maternal and Child Health University of Genova Italy; ^60^ School of Computing Newcastle University Newcastle upon Tyne UK; ^61^ Institute of Translational and Clinical Research Newcastle University Newcastle upon Tyne UK; ^62^ Department of Medicine, Royal Melbourne Hospital University of Melbourne Parkville Victoria UK; ^63^ Department of Neuropsychiatry Royal Melbourne Hospital Parkville Victoria Australia; ^64^ Department of Neurology Royal Melbourne Hospital Melbourne Victoria Australia; ^65^ Institute of Experimental Epileptology and Cognition Research University of Bonn Bonn Germany; ^66^ Cognitive Science Department School of Informatics, Xiamen University Xiamen China; ^67^ Department of Psychiatry University of California San Diego La Jolla California USA

**Keywords:** covariance, deep learning, DTI, event‐based modeling, gene expression, genetics, imaging, MRI, quantitative, rsfMRI

## Abstract

Epilepsy is a common and serious neurological disorder, with many different constituent conditions characterized by their electro clinical, imaging, and genetic features. MRI has been fundamental in advancing our understanding of brain processes in the epilepsies. Smaller‐scale studies have identified many interesting imaging phenomena, with implications both for understanding pathophysiology and improving clinical care. Through the infrastructure and concepts now well‐established by the ENIGMA Consortium, ENIGMA‐Epilepsy was established to strengthen epilepsy neuroscience by greatly increasing sample sizes, leveraging ideas and methods established in other ENIGMA projects, and generating a body of collaborating scientists and clinicians to drive forward robust research. Here we review published, current, and future projects, that include structural MRI, diffusion tensor imaging (DTI), and resting state functional MRI (rsfMRI), and that employ advanced methods including structural covariance, and event‐based modeling analysis. We explore age of onset‐ and duration‐related features, as well as phenomena‐specific work focusing on particular epilepsy syndromes or phenotypes, multimodal analyses focused on understanding the biology of disease progression, and deep learning approaches. We encourage groups who may be interested in participating to make contact to further grow and develop ENIGMA‐Epilepsy.

## 1 INTRODUCTION

ENIGMA‐Epilepsy, the epilepsy working group of ENIGMA, was officially launched at The Royal Society, London in March 2015 by Christopher Whelan (USC) and Sanjay Sisodiya (UCL) at a meeting of the International League Against Epilepsy Consortium on Complex Epilepsies (https://www.ilae.org/guidelines/complex-epilepsies). The project has continued to grow since its inception. It is now jointly led by Carrie McDonald, Christopher Whelan, and Sanjay Sisodiya, and comprises 26 collaborating international centers. The original aims of the group are multiple and include: to create a worldwide network of epilepsy neuroimaging centers; to collect summary statistics on brain shape, brain volume and white matter connectivity from thousands of people with epilepsy and thousands of neurologically healthy controls; to compare and contrast these measures in affected/unaffected groups and, accordingly, illustrate possible differences between the two; to identify structural differences between the major forms of epilepsy and major types of seizure; and to develop collaborations and infrastructure for future analyses. ENIGMA‐Epilepsy was formed with the intention of applying the idea that had proven effective in other ENIGMA projects, based on large‐scale, world‐wide collaborative efforts merging different types of biological data set (e.g., MRI, genetic, EEG) to deepen understanding of neurobiology in health and disease. Building on prior models in neuropsychiatric diseases, such as schizophrenia (SCZ) and major depressive disorder (MDD), and in imaging‐genetic studies in healthy subjects, ENIGMA‐Epilepsy adopted the template from these existing projects within the ENIGMA umbrella. We adapted the existing memorandum of understanding (MoU), and replicated pathways for development and consolidation of the consortium.

Prior to ENIGMA‐Epilepsy, there was a rich existing literature on MRI in epilepsy, with a number of productive groups working around the world. MRI had already proven hugely valuable in clinical and research application in the epilepsies. However, most published studies were only based on modestly sized samples, with typically fewer than 100, and often fewer than 50, participants included. Unsurprisingly, the limited power from such studies meant that findings from different studies of the same problem were often contradictory or inconclusive. In addition, new methods, many developed or championed by ENIGMA, were successfully promoting the formal joint analysis of disparate data sets, whether of the same type spread across different centers (e.g., in Australia vs. Canada), or of different types (e.g., MRI vs. genome‐wide SNP data). In this context, the formation of ENIGMA‐Epilepsy was a natural step forward.

**FIGURE 1:**
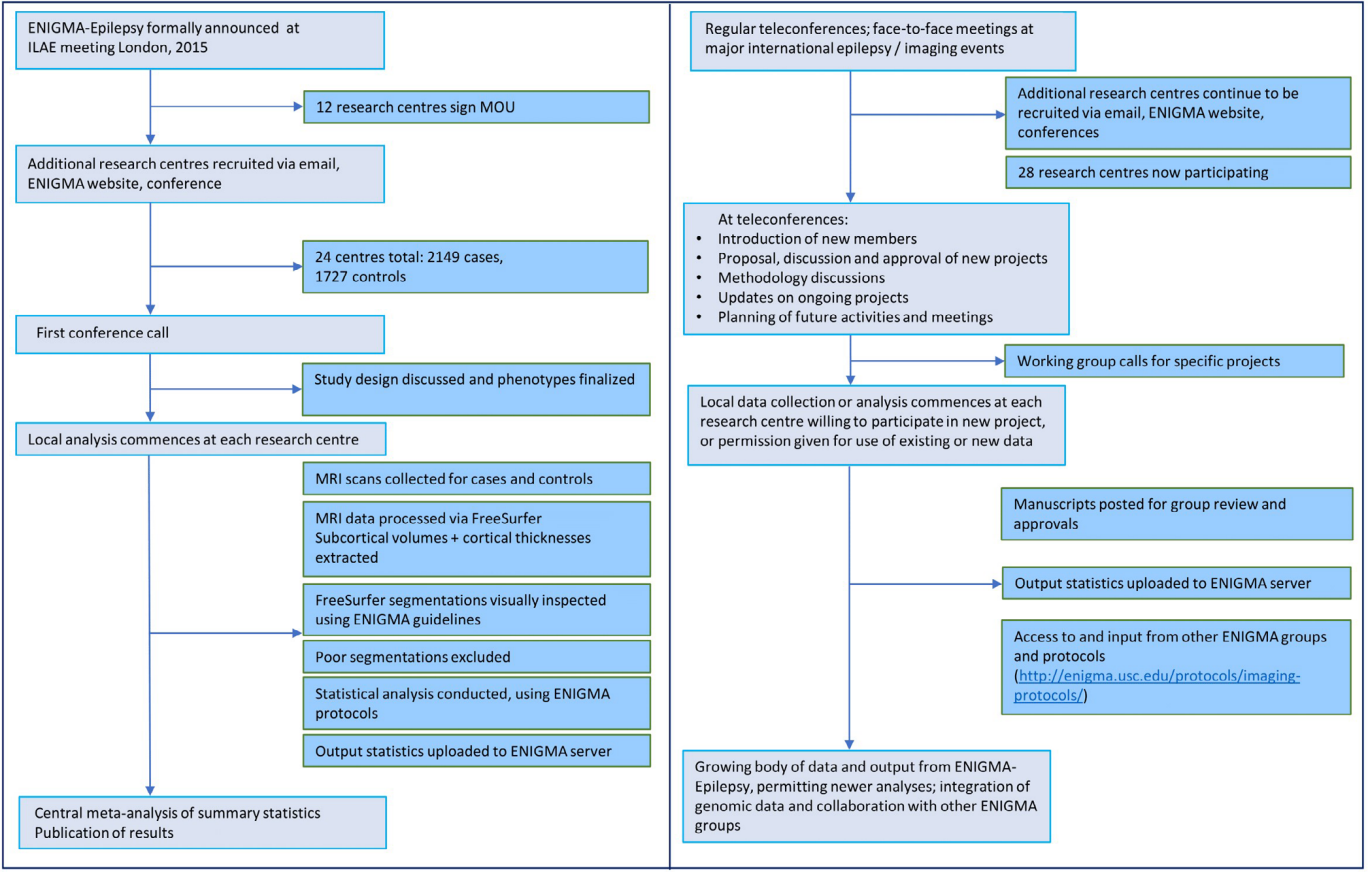
Organizational diagram of ENIGMA‐Epilepsy. The flowchart on the left shows the group set up, illustrating source of data used in subsequent studies. The current workflow is illustrated on the right. ILAE, International League Against Epilepsy; MOU, Memorandum of Understanding

Here we describe projects completed or still in progress within ENIGMA‐Epilepsy, and our hopes for its future. The structure and workflow is illustrated in Figure 1. We provide contact details and invite any group with relevant resources and appropriate intent interested in joining the consortium to get in contact.

## 2 PROJECTS AND PUBLICATIONS

The first projects within ENIGMA‐Epilepsy were analyses of structural and diffusion MRI data without any genetic, cognitive, or clinical outcome data. These projects allowed the consortium to organize itself and establish a modus operandi, and were based on distributed analyses, with each center applying a shared processing protocol locally to its own data (from people with epilepsy and healthy controls scanned on the same platform), with no exchange of raw image (i.e., DICOM) data, and subsequent central collation and meta‐analysis and mega‐analysis of the processed (i.e., region‐of‐interest) outputs. These methods have been of proven utility, and have overcome platform‐related issues in cross‐center analysis, as demonstrated by the extensive publication record of ENIGMA as a whole (Thompson et al., 2020).

The simplicity and effectiveness of this strategy shaped ENIGMA‐Epilepsy into an operational grouping, and has already led to measurable outcomes. The first three projects were a worldwide study of brain structure in epilepsy, a similar study of diffusion MRI data, and the first consortium approach to perform joint analysis of multiple types of data to explore mechanisms underlying findings emerging from the initial studies that were based solely on imaging data. Building on these three studies, members were encouraged to propose secondary projects, bringing new ideas, methods, and resources to bear on the collated imaging data set.

### 2.1 The first ENIGMA‐Epilepsy study: A worldwide study of brain structural changes in epilepsy using quantitative structural MRI

By late summer of 2015, the working group had organized its first conference calls and agreed upon a broad study design, which was to apply ENIGMA's standardized image processing and meta‐analysis protocols across a large network of research groups, with the broad goal of identifying robust patterns of gray matter alterations across different forms of commonly‐occurring epilepsy, addressing prior inconsistencies from smaller‐scale studies in the field.

For this inaugural project, the working group set out to compare data from neurologically healthy individuals (total *n* = 1,727) to four broad epilepsy subtypes, including (a) temporal lobe epilepsies (TLE) with left mesial temporal sclerosis (*n* = 415), (b) TLE with right mesial temporal sclerosis (n = 339), (c) genetic generalized epilepsies (GGE; *n* = 367), and (d) all other epilepsies (*n* = 1,026), in addition to a fifth collection of all epilepsies in aggregate (*n* = 2,149). Other epilepsy syndromes, such as frontal lobe or occipital lobe epilepsies, were not considered due to their limited presence across the majority of research centers. In total, the study included 24 case–control samples, recruited across 14 countries, aged 18–55 years. All syndromic epilepsy classifications were conducted by clinical epilepsy specialists, using International League Against Epilepsy (ILAE) systems, with prespecified inclusion and exclusion criteria (details in Whelan et al., 2018, Tables S1 and S2). These classifications were carried through to subsequent studies.

T1‐weighted MRI scans were acquired using a variety of platforms at each research center (the majority comprising three Tesla Siemens or Philips platforms, applying magnetization‐prepared rapid gradient echo [MP‐RAGE] sequencing; details provided in Whelan et al., 2018, Table S3). At each of the 24 sites, a trained neuroimaging analyst conducted quality assessment of MRI scans using the ENIGMA protocols, which included an initial segmentation step conducted using FreeSurfer v5.3, whereby 12 subcortical brain regions and 64 cortical regions were extracted from the T1‐weighted image, followed by a combination of automated outlier detection and detailed visual inspection for each segmentation. MRI scans from each of the five predefined epilepsy groups were then compared with healthy controls using a series of linear regressions in R, adjusting for age, sex, and intracranial volume. A series of linear regressions testing the association between brain measures and age at onset of epilepsy, as well as overall duration of epilepsy, was also conducted at each center. Summary statistics from these analyses (including Cohen's D effect sizes and regression beta coefficients) were transferred from each research center to a central analysis laboratory in the Imaging Genetics Center at the University of Southern California, where they were pooled across sites using a random‐effects meta‐analysis model. Given the number of comparisons conducted as part of the study (336 in total), the typical statistical significance threshold of *p* < .05 was Bonferroni‐adjusted to *p* < 1.49 × 10^−4^.

**FIGURE 2:**
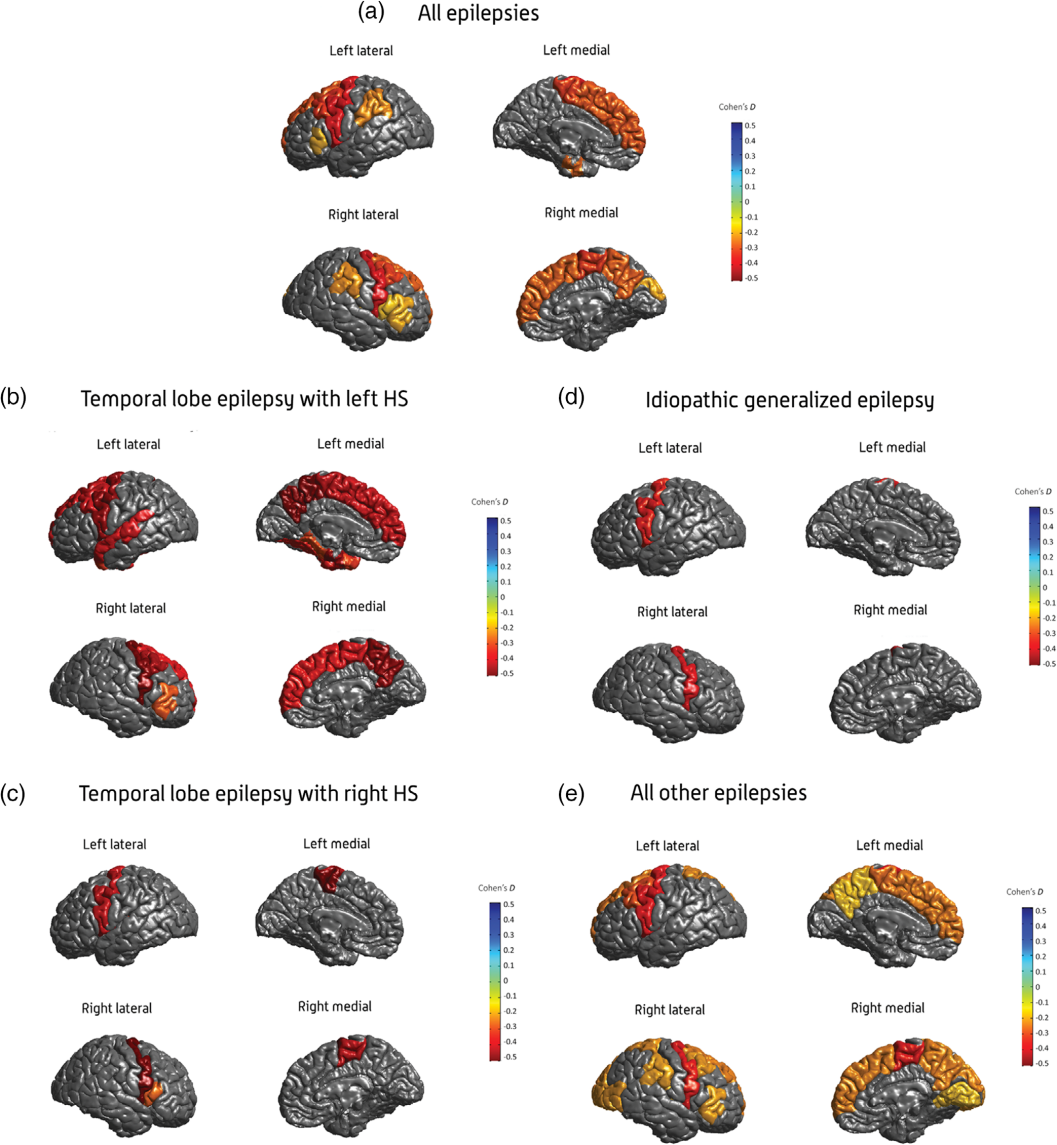
An illustration of results from the first ENIGMA‐Epilepsy study, of brain structural changes in epilepsy using quantitative structural MRI. Cohen's d effect size estimates for case–control differences in cortical thickness, across the (a) all‐epilepsies, (b) mesial temporal lobe epilepsies with left hippocampal sclerosis (c) mesial temporal lobe epilepsies with right hippocampal sclerosis, (d) idiopathic generalized epilepsies, and (e) all‐other‐epilepsies groups. Cohen's d effect sizes were extracted using multiple linear regressions, and pooled across research centers using random‐effects meta‐analysis. Cortical structures with *p*‐values <1.49 × 10^−4^ are shown in heatmap colors; strength of heat map is determined by the size of the Cohen's d (d < 0 = blue, d > 0 = yellow/red). HS, hippocampal sclerosis. From Whelan et al., 2018

The study revealed a series of robust structural brain alterations, both within and across epilepsy syndromes (Figure 2) in section 1. As expected, MTLEs showed profound volume reductions in the hippocampus ipsilateral to seizure onset (d < −1.7; *p* < 1.4 × 10^−19^). When compared with healthy controls, left temporal lobe epilepsy (TLE) appeared to show a more widespread and bilateral distribution of extrahippocampal gray matter differences than did right TLE, resolving prior inconsistent findings in smaller‐scale studies. The right thalamus (Cohen's d = −0.24 to −0.73; *p* < 1.49 × 10^−4^), and the bilateral precentral gyri (d = −0.34 to −0.52; *p* < 4.31 × 10^−6^) showed evidence of gray matter alterations across all five epilepsy subgroups when compared with healthy controls, highlighting prior observations that the thalamus may serve as a “hub” within a distributed function network that becomes disrupted during epileptogenesis, and re‐emphasizing how focal seizures can lead to persistent, potentially damaging hyperexcitability in distant cortical regions, particularly the ipsilateral motor cortices (Hamer et al., 2005). Duration of epilepsy showed a strong correlation with multiple subcortical volume and cortical thickness measures, in keeping with recent findings that epilepsy may be associated with progressive cortical atrophy (Galovic et al., 2019); however, given that, our large study had a cross‐sectional design, these findings strongly emphasize the need for more large‐scale, longitudinal neuroimaging investigations of seizure disorders. Bilateral enlargement of the amygdala, a finding previously—albeit inconsistently—reported across “nonlesional” forms of TLE, was seen in the “all other epilepsies” subgroup, warranting further investigation of TLE with amygdala enlargement as its own distinct syndrome, potentially with an autoimmune cause (Malter et al., 2016).

These findings from ENIGMA‐Epilepsy's first major study were discussed during an invited platform presentation at the annual meeting of the Organization for Human Brain Mapping in 2016, and subsequently published in the journal *Brain* (Whelan et al., 2018). This initial project solidified a framework for future large‐scale investigations of the epilepsies, with several in vitro, in vivo, and postmortem follow‐up investigations currently ongoing, as outlined below.

### 2.2 White matter abnormalities across different epilepsy syndromes in adults

Following the launch of the structural MRI study, efforts within the consortium shifted toward meta‐analysis of DTI data. Our scientific premise was that epilepsy is a network disorder with widespread white matter injury to regions that extend far beyond the identified seizure focus with patterns that may be unique to each syndrome. We elected to maintain a similar approach to the analysis followed in the structural MRI study. However, there was consensus that further dividing the epilepsy syndromes would be informative. As a result, patients with TLE were divided into those with and without hippocampal sclerosis (HS) given prior evidence that these patient groups may have different epileptogenic networks and harbor different patterns of white matter loss (Mueller et al., 2009; Zaveri, Duckrow, De Lanerolle, & Spencer, 2001). Our final sample consisted of 1,069 healthy controls and 1,466 patients with epilepsy, including mesial TLE, nonlesional TLE (TLE‐NL), GGE, and nonlesional extratemporal epilepsy (ExE), making this the largest epilepsy DTI imaging study to date. As a secondary analysis, we compared the effect size estimates obtained in our aggregate group of epilepsy patients to those obtained by other ENIGMA working groups, including MDD, SCZ, bipolar disorder (BPD), and 22q11 syndrome.

We initially attempted the meta‐analytic approach previously used in ENIGMA‐MDD (van Velzen et al., 2019) and ENIGMA‐SCZ (Kelly et al., 2018). However, this strategy was not ideal for our patient data set, which included many different syndromes across many sites. Although the contrasts of “all patients in aggregate” versus controls produced robust and meaningful results, the syndrome‐specific results did not seem biologically plausible, likely due to the small number of participants per syndrome at many sites. Therefore, we instead pooled all the data together in a *mega*‐analysis—an approach that aggregates individual‐participant data across sites. Compared with meta‐analyses, mega‐analysis uses a more exact likelihood specification, which avoids the assumptions of within‐study normality and known within‐study variances, such as sites with few subjects, rare diagnoses, and/or unbalanced sample size between controls and patients (Burke, Ensor, & Riley, 2017). This approach also boosts statistical power and provides greater control of confounders at the individual subject level (Debray, Moons, Abo‐Zaid, Koffijberg, & Riley, 2013). Mega‐analyses are also robust to missing data and do not require imputation or removal of entire subjects when single data points are missing. The main reason for missing values in this data set were the removal of extreme outliers (greater than ±3*SD*) which was between 1 and 5 regions of interest per site per diffusion parameter.

Aggregating DTI data across sites also requires careful data harmonization given the known variability in diffusion parameters across scanner platforms. Following the approach utilized in two prior mega‐analyses, we applied a novel batch‐effect correction tool, ComBat, to adjust for scanner/site specific variations in diffusivity measures (Fortin et al., 2017; Villalón‐Reina et al., 2019; Zavaliangos‐Petropulu et al., 2019). ComBat uses an empirical Bayes framework to improve the variance of the parameter estimates, assuming that all regions of interest share the same common distribution. Inspection of the corrected data suggested that ComBat successfully harmonized across the sites/scanner instances while maintaining syndrome‐specific features that were biologically plausible.

**FIGURE 3:**
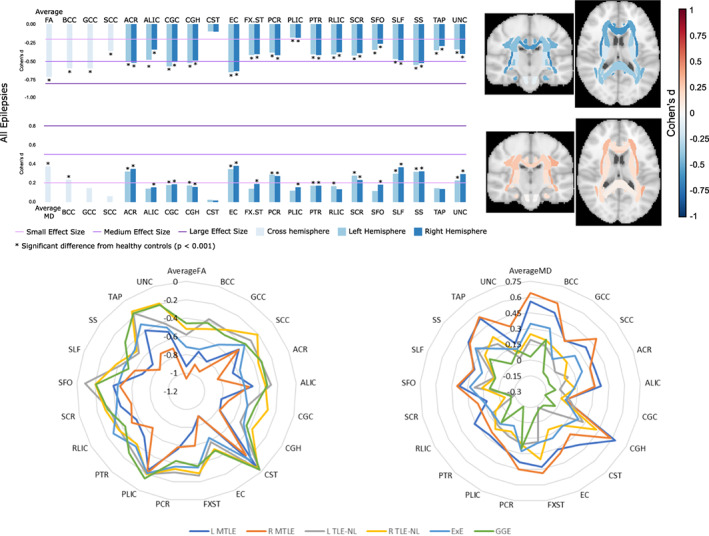
White matter abnormalities across different epilepsy syndromes in adults. Top: FA and MD across all patients with epilepsy. BCC, body of corpus callosum, GCC, genu of corpus callosum; SCC, splenium of corpus callosum; ACR, anterior corona radiata; ALIC, anterior limb of internal capsule; CGC, cingulum (cingulate gyrus); CGH, cingulum (hippocampal); CST, corticospinal tract; EC, external capsule; FX.ST, fornix (stria terminalis); PCR, posterior corona radiata; PLIC, posterior limb of internal capsule; PTR, posterior thalamic radiation; RLIC, rentrolenticular part of internal capsule; SCR, superior corona radiata; SFO, superior fronto‐occipital fasciculus; SLF, superior longitudinal fasciculus; SS, sagittal stratum; TAP, tapetum; UNC, uncinate. Bottom: Radar plots of FA (left) and MD (right) across epilepsy syndromes

Findings from our harmonized mega‐analysis revealed microstructural abnormalities across major association, commissural, and projection fibers in patients with epilepsy (Figure 3). In our aggregate analysis of all patients, lower FA was observed in most white matter pathways, especially in the genu and body of the corpus callosum (CC), cingulum (CING) and external capsule (EC). Syndrome‐specific FA/MD differences were most pronounced in patients with MTLE, with large effect size differences in the ipsilateral parahippocampal cingulum and EC, and small to medium effect sizes across most other tracts. TLE‐NL showed a similar ipsilateral greater than contralateral pattern, but with smaller effect size differences compared with MTLE. Patients with GGE and ExE demonstrated the most pronounced effect size differences in the CC and EC (FA), and anterior corona radiata bilaterally (FA/MD). Earlier age of seizure onset and longer disease duration were associated with altered white matter microstructure in patients with right and left MTLE. As a whole group, patients showed white matter perturbation in anterior, midline fibers, while the severity was different across the epilepsy syndromes. We observed a similar pattern of results in epilepsy patients to those observed in MDD, SCZ, and BPD. We believe that these data can further inform our understanding of epilepsy as a white matter network disorder with both nonspecific (i.e., shared across brain disorders) and syndrome‐specific microstructural alterations. A next step for our consortium will be to test whether white matter alterations identified in our mega‐analysis can predict postoperative seizure outcome or to discriminate patients who are drug‐responsive from those who are drug‐resistant. These follow‐up studies will also explore age and sex effects more comprehensively. Finally, there is interested in combining the T1‐derived structural data with the DTI‐derived diffusion data to investigate the multimodal interplay of brain structure and connectivity in epilepsy. The current study is under review, with a preprint available at https://www.biorxiv.org/content/10.1101/2019.12.19.883405v1.

### 2.3 Gene expression and brain maps

As outlined above, the structural MRI analysis revealed widespread differences in cortical thickness in people with epilepsy compared with healthy controls. Although many cortical regions appeared to be vulnerable (i.e., showed significantly thinner cortices) other regions appeared to be unaffected by (protected from) the disease. In this follow‐up project, we integrated these imaging results with the detailed brain‐wide gene expression atlas generated by the Allen Institute of Brain Science (Hawrylycz et al., 2012), to investigate whether there was a molecular biological signature that explains the regional vulnerability. Briefly, the gene expression atlas combines data from six donors and provides the expression level for each of more than 21,000 genes at 3,702 locations in the cortex, subcortical regions, and the cerebellum.

**FIGURE 4:**
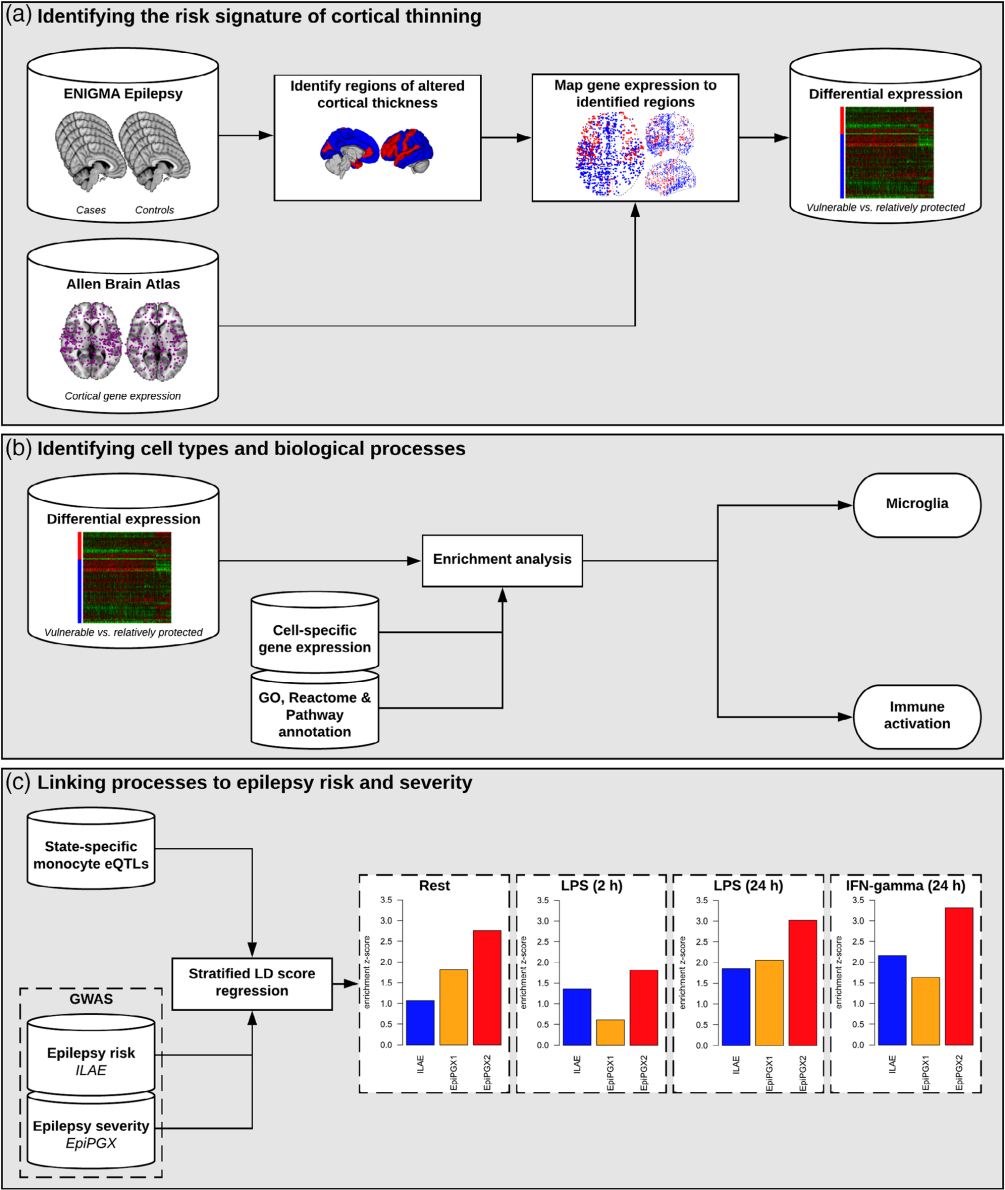
Gene expression and brain maps. The study strategy leading to implication of microglia in cortical thinning. eQTL, expression quantitative trait loci; ILAE, International League against Epilepsy; EpiPGX, Epilepsy Pharmacogenomics: delivering biomarkers for clinical use project, www.epipgx.eu; LD, linkage disequilibrium; LPS, lipopolysaccharide

Our analysis found that over 2,500 genes exhibited higher expression in vulnerable regions compared with protected regions (Altmann et al., 2018). Detailed analysis of these overexpressed genes showed enrichment for genes related to microglia and inflammation, which suggested that vulnerable regions might exhibit a higher density in microglia. By further studying postmortem brain tissue from individuals with nonlesional epilepsy, lesional epilepsy, and nonepilepsy controls, we found higher microglia density across all tested regions in samples from people with epilepsy compared with controls. Thus, these results were consistent with the view that there was an over‐representation in brain tissue from people with chronic epilepsy and that such microglial responses may occur in a regionally specific manner. Finally, we turned to a mouse model of acquired epilepsy (Iori et al., 2017) to establish a causal link between microglia activity and cortical thinning. The experiments revealed cortical thinning in the entorhinal cortex in epileptic mice compared with control mice, which was partially due to reduced neuronal cell density and average neuronal size. Importantly, appropriately timed microglial depletion (through treatment with PLX3397) could prevent or substantially reduce the amount of observed cortical thinning. The rescue was mainly acting on neuronal cell density (neuronal size changes were not rescued). Thus, taken together these findings incriminate potentially modifiable microglial activation states in cortical thinning in the common human epilepsies, and illustrate the value of the MRI data set available in ENIGMA‐Epilepsy (Figure 4): no other tool (PET, histopathology) would have generated such a large‐scale and well‐powered initial data set to drive these follow‐on analyses. This study is ongoing.

### 2.4 Secondary proposals

In addition to the main projects listed above, group members proposed their own projects making the best use of the gathered data. The current projects are listed below. Further projects are anticipated.

#### 2.4.1 Structural correlates of mild mesial temporal lobe epilepsy with and without hippocampal sclerosis

TLE is the most common form of focal epilepsy in adulthood. When seizures start in the internal portion of the temporal lobe, the syndrome is called mesial TLE (MTLE), which accounts for 80% of TLE cases. More than 30% of patients with MTLE do not respond to anti‐epileptic medications, whereas a mild form of MTLE (mMTLE) has been recently described, which is characterized by seizure onset in adulthood, unremarkable past medical history, viscero‐sensory auras, and long‐term seizure freedom (>24 months) with or without antiepileptic medication (Labate et al., 2011). Approximately one third of patients with mMTLE have MRI evidence of hippocampal sclerosis (HS), which was previously considered a hallmark of refractoriness. Mild MTLE represents an important opportunity to better delineate the biological substrates underlying the epileptic syndrome itself, and the ENIGMA‐Epilepsy project has provided a unique chance to carry on this investigation over a very large cohort of subjects. Adult‐onset mMTLE often provides few clues to suspect delayed appearance of refractoriness. Very recently, a longitudinal study has shown the long‐term outcome of patients with mMTLE over a mean follow‐up of 12 years (Labate et al., 2016). Over this period, 25% of the patients, who all had mMTLE at time of enrolment, eventually developed refractoriness. Survival analysis showed that mMTLE patients carrying radiological evidence of HS since recruitment have a three times higher likelihood of becoming refractory later on in life than those without HS.

In the light of these findings, and with the support of the ENIGMA‐Epilepsy Working Group, we are using locally‐processed T1‐weighted MRI data from multiple centers to investigate differences in mMTLE patients divided on the basis of the presence/absence of HS, since this has been found to be the major risk factor for development of refractoriness. This provides an unprecedented chance to study this relatively benign syndrome on a larger scale, to test the hypothesis that people with mMTLE with HS may present different patterns of subcortical atrophy and cortical thinning compared with patients without HS and to healthy controls. While the first stage of the project has taken into account only structural data, in the future we aim to integrate data from the DTI study of ENIGMA‐Epilepsy, to investigate potential white matter correlates specific to mMTLE with HS.

#### 2.4.2 Structural covariance networks and network‐based atrophy modeling

The adoption of mathematical techniques to study complex systems in neuroimaging has led to a shift in understanding healthy and diseased brains away from a focus on individual regions, and toward approaches highlighting the importance of large‐scale brain networks (Bassett & Sporns, 2017; Bullmore & Sporns, 2009). Indeed, network neuroscience methods have become increasingly important to study brain development as well as aging, to capture typical inter‐individual variations in structural and functional measures, and to understand the impact of disorders on whole‐brain structure and function (Fornito, Zalesky, & Breakspear, 2015; Stam, 2014).

Our knowledge of epileptic disorders has undergone a similar paradigm shift, with focal epilepsies being increasingly conceptualized as conditions affecting large‐scale cortical and subcortical networks (Bernhardt, Bonilha, & Gross, 2015; Engel Jr et al., 2013; Gleichgerrcht, Kocher, & Bonilha, 2015; Richardson, 2012; Tavakol et al., 2019). Using measures of brain morphology, such as cortical thickness or subcortical volumetry, a series of studies has investigated inter‐regional structural networks by analyzing correlational patterns in morphological measures across patient populations suffering from both focal and generalized seizures. These structural covariance analyses tap into the coordinated morphology of different brain areas, with high covariance being often observed in regions also undergoing similar maturational trajectories during development and in regions participating in similar functional networks (Alexander‐Bloch, Raznahan, Bullmore, & Giedd, 2013). In epilepsy, initial evidence on the capacity of covariance analyses to tap into network‐level disease effects has come from previous work focusing on the covariance of specific regions, seeding from nodes in the mesial temporal lobe in drug‐resistant TLE (Bernhardt et al., 2008; Mueller et al., 2009) or from the thalamus in patients with GGE (Buckner et al., 2009). These studies have often demonstrated perturbations in the covariance patterns of such disorder‐associated regions. Other studies have assessed inter‐regional covariance more systematically, aggregating covariance patterns between multiple seeds to derive large‐scale network representations (Bernhardt, Chen, He, Evans, & Bernasconi, 2011; Yasuda et al., 2015). These networks of coupled cortical and subcortical morphology have frequently been analyzed using graph theoretical measures to shed light onto topological properties and organizational principles (Rubinov & Sporns, 2010). In patients with drug‐resistant epilepsy, several covariance analyses have been published to date, complementing work based on resting‐state fMRI (rsMRI) and DTI tractography (Bernhardt et al., 2019; Bonilha et al., 2015; Liu, Chen, Beaulieu, & Gross, 2014; van Diessen et al., 2014). Although such studies report heterogeneous findings, the majority of findings indicate that “focal” epilepsies are paradoxically associated with marked reconfigurations of structural network topology, often involving increases in path length (indicating reduced global efficiency) and clustering (indicating tighter cohesion of local communities) TLE patients relative to controls.

In addition to covariance analyses, the cortical morphological feature data and specifically disorder‐related atrophy maps produced during the first phase of the ENIGMA‐Epilepsy can also be related to network‐level structural, functional, and diffusion measures seen in healthy populations. In several neurodegenerative and neuropsychiatric conditions, such paradigms have proven useful to link cross‐sectional patterns of structural compromise with normative network organization, for example in the context of building models of regional susceptibility. Assessing different neurodegenerative and psychiatric disorders has for example indicated that network hubs (i.e., regions with many connections) are generally susceptible to marked atrophy in many disorders (Buckner et al., 2009; Crossley et al., 2014), potentially due to their high metabolic activity, their high plasticity, and participation across multiple networks. Complementing such “nodal stress” models, a series of studies has also assessed spatial similarity between disorder‐specific atrophy maps and different connectivity measures centered on specific seeds, in order to identify “epicenters” of network‐level structural compromise (Zeighami et al., 2015; Zhou, Gennatas, Kramer, Miller, & Seeley, 2012).

The ENIGMA‐Epilepsy data set provides the added opportunity of studying structural covariance networks across syndromes (e.g., TLE, GGE, other epilepsies) in the largest cohort of epilepsy patients available to date. In addition, structural atrophy and covariance maps can be combined with normative connectivity data in order to yield network‐level support for pathological epicenters in the common epilepsies.

#### 2.4.3 Disease progression modeling in epilepsy

The first publication by the ENIGMA‐Epilepsy working group revealed widespread differences in cortical thickness as well as volumes of subcortical structures in people with epilepsy compared with controls (Whelan et al., 2018). It remains unclear whether these differences represent developmental differences (i.e., reduced cortical thickness and volumetric differences are more prevalent in people who are more likely to develop epilepsy) or are the result of atrophy as the disease progresses. The data presented by Whelan et al. (2018) showed negative correlations between cortical thickness and subcortical volumes and disease duration, suggesting progressive atrophy in the common epilepsies. However, the gold standard to establish causality between disease and atrophy are longitudinal studies that quantify the loss of gray matter between two or more MRI scans. Recently, such a study comprising 190 people with epilepsy and 141 controls showed widespread cortical thinning exceeding normal aging effects (Galovic et al., 2019). Taken together there is evidence supporting the progressive nature of gray matter loss.

A complementary approach to the longitudinal study design are disease progression models, which, broadly speaking, can leverage cross‐sectional data to infer the underlying longitudinal model of change (Oxtoby, Alexander,,, & EuroPOND consortium, 2017). One such approach is the Event Based Model (EBM), which was introduced in the domain of familial Alzheimer's disease and Huntington's disease (Fonteijn et al., 2012) and has by now found wide application in many brain disorders. Briefly, the idea behind the EBM is that if one biomarker (**A**) typically reaches an abnormal level before a second biomarker (**B**) reaches an abnormal level, then in a data set we expect to see many people who have abnormal levels of biomarkers **A** alone, or **A** and **B** together. However, we would expect to see few people with abnormal levels of biomarker **B** but normal levels of biomarker **A**. Given a data set, the EBM uses this reasoning across all subjects and biomarkers to reconstruct the most likely order in which biomarkers reach abnormal levels over the course of the disease. In the case of ENIGMA‐Epilepsy, volumes of subcortical structures and regional cortical thickness are treated as separate biomarkers. During the training phase, the EBM converts each subjects' biomarkers into probabilities of these biomarkers being abnormal; next, it evaluates possible biomarker orderings and seeks to find the ordering that maximizes the likelihood of the observed biomarker pattern in the entire data set. Stability of this ordering is assessed using cross validation. After the EBM has been trained, it provides the most likely ordering of biomarkers, which can improve understanding of the disease process, for example by uncovering that hippocampal atrophy precedes atrophy in the thalamus. More importantly however, a new subject can be objectively staged (i.e., the most likely stage is computed) using the trained EBM. For instance, the EBM is trained on 10 biomarkers, then, subjects can be assigned a stage from zero to 10, meaning that none or all biomarkers have reached abnormal levels, respectively. Practically, a stage of *k* means that the first *k* biomarkers (in the order estimated by the EBM) have reached abnormal level in the given subject. The inferred stage can then be linked in subsequent analyses to clinical markers of disease severity or prognosis.

Within this secondary project, we are applying the EBM to the collected structural MRI data with the aim to infer the order in which brain regions show abnormal values of gray matter in TLE, GGE, and other epilepsies. Of particular interest is the comparison between left and right TLE and whether the biomarker order in left TLE is the same as in right TLE, just with swapped laterality. Moreover, the project will investigate whether the EBM staging corresponds to duration of illness or markers of disease severity, and thus may be of clinical value.

#### 2.4.4 Resting state functional connectivity in people with TLE

TLE can be considered a disorder involving a discrete set of limbic and paralimbic brain regions involved in the generation and propagation of seizures. However, human and animal models have shown that numerous cortical and subcortical brain regions are involved in the condition. These findings support the view that seizure activity in focal epilepsy originates within intra‐hemispheric networks, which is particularly relevant in understanding impairments in higher order brain functions that commonly occur with the condition.

RsfMRI allows the identification of atypical functional connectivity within and between large‐scale brain networks and provides a unique opportunity to study dysfunctional brain architecture. Preliminary evidence from resting state studies suggests that the condition chronically alters activity in brain networks controlling basic functions such as cognition, attention, and emotion (Seeley et al., 2007; Buckner, Andrews‐Hanna, & Schacter, 2008; Harrison et al., 2008; Sheline, Price, Yan, & Mintun, 2010; van den Heuvel & Hulshoff Pol, 2010; Clemens et al., 2011; Cataldi, Avoli, & de Villers‐Sidani, 2013): these findings need to be validated in larger samples.

Through the ENIGMA‐Epilepsy working group, we have launched a multicenter study to identify atypical functional connectivity in resting‐state networks in people with TLE. This study will enable the validation of preliminary findings of network abnormalities reported in people with epilepsy, advance our understanding of disordered functional anatomy in TLE and provide insights into clinical manifestations of TLE that are currently unexplained.

#### 2.4.5 Using imaging and genetic data sets to explore the mechanisms underlying additional phenotypes in the epilepsies: Drug resistance and sudden unexpected death in epilepsy

In clinical epilepsy practice, there are many important phenotypic features that are unexplained. Amongst these are the phenomenon of drug resistance, with seizures that continue to occur despite treatment with appropriate antiseizure drugs: drug resistance occurs in about 30% of patients, and is associated with higher rates of comorbidities and premature mortality (Löscher, Potschka, Sisodiya, & Vezzani, In press). Sudden unexpected death in epilepsy (SUDEP) is a tragic outcome which affects an incidence of at least 1 per 1,000 patient years, with higher rates in people with uncontrolled generalized tonic–clonic seizures (Whitney & Donner, 2019). Neither important aspect of the epilepsies is currently preventable, nor fully understood. In SUDEP, brain structural and functional imaging abnormalities have been reported (Allen et al., 2017; Allen et al., 2019; Allen, Harper, Lhatoo, Lemieux, & Diehl, 2019; Wandschneider et al., 2015), but these are from comparatively small studies. ENIGMA‐Epilepsy offers the chance to study these aspects of epilepsy in larger numbers of patients, and both drug resistance and SUDEP are active areas of study in ENIGMA‐Epilepsy, with the added value that genetic data can also be merged with information from processed MRI data.

#### 2.4.6 Deep learning

Deep learning, a type of machine learning, can be broadly summarized as artificial intelligence (AI), which collectively offers the benefits of identifying patterns in complex data and out‐of‐sample prediction. It can provide some additional insights, typically if very large data sets are employed.

In the context of neuroimaging, patterns in complex data can be difficult for the human eye to fully grasp, particularly when multiple dimensions are present. For example, it is relatively straightforward to appreciate that the hippocampus may exhibit atrophy on T1 weighted images, hypometabolism on interictal nuclear medicine studies and high‐probability of being a generator of interictal discharges on high‐density EEG source reconstruction. However, it becomes more difficult to fully appreciate the complexity of multiple dimensions when broader networks are involved and do not fully overlap, or when there are more subtle abnormalities. In this context, AI offers the benefits of “filtering” this information and providing a summary of the concordant versus nonconcordant information from multiple modalities. This is particularly relevant when imaging modalities such as functional or structural connectivity are taken into account, since these can be somewhat difficult to visualize. AI can provide important decision support tools for the identification of clinical phenotypes.

Machine learning applied to epilepsy is still in its nascent stages, but there have been promising results from pilot studies suggesting that it can be sensitive to abnormalities related to the disease. For example, previous work has demonstrated high accuracy in discriminating the brains of people with TLE from those of healthy controls using support vector machine (SVM) based on microstructural abnormalities in the medial inferior aspect of the temporal lobes (Del Gaizo et al., 2017). Using multimodal imaging, Bennet et al. demonstrated that seizure laterality could be predicted with high accuracy in MRI‐positive and MRI‐negative cases of TLE based on hippocampal or temporal lobe information, respectively (Bennett et al., 2019). Importantly, Kamiya demonstrated that DTI structural brain connectomes (which are invisible to the human eye) could be accurately used for lateralization of TLE (Kamiya et al., 2016). These studies underscore a few important points:

1. Conventional machine learning could be helpful for detecting invisible patterns in TLE that could help to lateralize seizure onset and possibly predict clinical outcomes (e.g., neuropsychological function) (Bennett et al., 2019; Del Gaizo et al., 2017; Frank et al., 2018; Kamiya et al., 2016; Munsell et al., 2019).
2. The findings so far have been mostly restricted to TLE. Arguably, phenotyping other forms of epilepsy may be of greater importance since these are the most challenging cases, for which computer‐aided diagnosis may be most needed.
3. While conventional machine learning is a powerful form of AI, deep learning offers a wider range of applications and potentially better accuracy. Given their high parametric complexity, these algorithms may perform particularly well when leveraging big data sets.

ENIGMA‐Epilepsy provides a large representative multimodal imaging data set across multiple centers, being thus optimal for the application of deep learning in epilepsy. The limitations related to sample size and representativeness of data are minimized by the collaborative environment and by the breadth of data. We recently obtained NIH support for a pilot project (NINDS R21) to evaluate whether deep learning is better in classifying TLE in comparison with conventional machine learning, as well as whether deep learning can successfully predict seizure outcomes following epilepsy surgery. After this initial step is complete, future directions by ENIGMA‐Epilepsy will include: (a) leveraging complex connectome‐based neural network patterns, arguably reaching at the core of pathophysiology of epilepsy, and leveraging deep learning ability to segment features into subnetworks; (b) assessing convolutional neural networks with unprocessed T1 weighted data or K‐space data; and (c) the expansion of these approaches to extratemporal epilepsy Figures 1, 2, 3, 4.

#### 2.4.7 Structural changes at onset of illness

Diagnosis and management of patients who experience an event with transient neurological deficit or loss of consciousness for the first time is a challenging task, especially if no structural epileptogenic lesion can be detected. Routine diagnostic methods to identify patients with new‐onset epilepsy are of limited sensitivity. A prospective multicenter study based in Switzerland, “Predict and Monitor Epilepsy after a First Seizure: The Swiss First Study” aims to determine EEG and MRI‐based biomarkers that identify network abnormalities characterizing people with epilepsy. Neuroimaging research has mainly focused on patients with a long history of epilepsy (Crocker, Pohlmann‐Eden, & Schmidt, 2017). With a mean disease duration of 17.4 years (± 12.0 years), the ENIGMA‐Epilepsy cohort comprises predominantly people with long‐standing epilepsy (Whelan et al., 2018). It remains undetermined if and which structural abnormalities constitute pre‐clinical cortical reorganization caused by a predisposition to epilepsy or whether such abnormalities develop as a consequence of the ongoing illness (see also Section 2.4.3).

To address this question, a sub‐analysis of the ENIGMA‐Epilepsy data set in patients with short duration of the disease is being executed within this secondary project. People will be stratified into cohorts of recent‐onset epilepsy (illness duration ≤ 2 years), short illness duration (duration ≤ 5 years) and chronic epilepsy (duration >5 years). Despite the strict restrictions in the first two subgroups, the group sizes reachable via the ENIGMA‐Epilepsy collaboration are expected to be much larger than possible in single‐center studies. We will explore differences between these three cohorts and matched healthy control groups. This sub‐analysis of the retrospectively‐collected ENIGMA‐Epilepsy structural MRI data set might generate new hypotheses for people with new‐onset epilepsy paralleling the prospective Swiss First study.

## 3 FUTURE PLANS

Membership of ENIGMA‐Epilepsy continues to grow: indeed, we invite researchers prompted to join our consortium as a result of this article to make contact as detailed below. Methods development within ENIGMA and across its constituent members provides tools for additional analyses of existing and new data. ENIGMA‐Epilepsy also intends to move to a centralized approach that involves sharing raw data, subject to the necessary governance structures, permitting formal mega‐analyses, as have a number of other groups in ENIGMA.

## CONTACT DETAILS

Those interested are invited to contact the coordinators.

The Memorandum of Understanding is available at: http://enigma.ini.usc.edu/ongoing/enigma-epilepsy/join-enigma-epilepsy/

## DISCLOSURES

N. J. and P. M. T. are partially funded by Biogen Inc, for research unrelated to the contents of this manuscript. N. K. F. has received honoraria from Bial, Eisai, Philips/EGI, UCB. M. S. holds shares in Epilog. D. J. S. has received research grants and/or consultancy honoraria from Lundbeck and Sun. C. D. W. is an employee of Biogen Inc.

## Data Availability

Data sharing is not applicable to this article as no new data were created or analyzed in this study.
